# National Survey of Oral/Dental Conditions Related to Tobacco and Alcohol Use in Mexican Adults

**DOI:** 10.3390/ijerph110303169

**Published:** 2014-03-17

**Authors:** Carlo Eduardo Medina-Solís, América Patricia Pontigo-Loyola, Eduardo Pérez-Campos, Pedro Hernández-Cruz, Leticia Ávila-Burgos, Martha Mendoza-Rodríguez, Gerardo Maupomé

**Affiliations:** 1 Academic Area of Dentistry of Health Sciences Institute at Autonomous University of Hidalgo State, Pachuca, Hidalgo 42160, Mexico; E-Mails: americap@uaeh.edu.mx (A.P.P.-L.); romen65@hotmail.com (M.M.-R.); 2 Research Centre in Medical and Biological Sciences, School of Medicine and Surgery, Autonomous University “Benito Juarez” of Oaxaca, Oaxaca 68020, Mexico; E-Mails: perezcampos123@yahoo.es (E.P.-C.); fuegoblanco136@yahoo.com.mx (P.H.-C.); 3 Biochemistry Unit ITO-UNAM, Oaxaca 68030, Mexico; 4 Health Systems Research Centre, National Institute of Public Health, Cuernavaca, Morelos 62100, Mexico; E-Mail: lavila@correo.insp.mx; 5 School of Dentistry, Indiana University/Purdue University at Indianapolis, Indianapolis, IN 46202, USA; E-Mail: gmaupome@iupui.edu; 6 The Regenstrief Institute, Inc. Indianapolis, IN 46202, USA

**Keywords:** oral health, epidemiology, smoking, alcohol, adults

## Abstract

Oral diseases are a major burden on individuals and health systems. The aim of this study was to determine whether consumption of tobacco and alcohol were associated with the prevalence of oral/dental problems in Mexican adults. Using data from the National Performance Evaluation Survey 2003, a cross-sectional study part of the World Health Survey, dental information from a representative sample of Mexico (*n* = 22,229, *N* = 51,155,740) was used to document self-reported oral/dental problems in the 12 months prior to the survey. Questionnaires were used to collect information related to sociodemographic, socioeconomic, and other risk factors. Three models were generated for each age group (18–30, 31–45 and 46–98 years). The prevalence of oral/dental conditions was 25.7%. Adjusting for sex, schooling, socioeconomic position, diabetes, and self-reported health, those who used tobacco (sometimes or daily) (OR = 1.15, *p* = 0.070; OR = 1.24, *p* < 0.01; and OR = 1.16, *p* < 0.05, for each age group respectively) or alcohol (moderate or high) (OR = 1.26, *p* < 0.001; OR = 1.18, *p* < 0.01 and OR = 1.30, *p* < 0.001, for each age group respectively) had a higher risk of reporting oral/dental problems. Because tobacco and alcohol use were associated with self-reported oral/dental problems in one out of four adults, it appears advisable to ascertain how direct is such link; more direct effects would lend greater weight to adopting measures to reduce consumption of tobacco and alcohol for the specific purpose of improving oral health.

## 1. Introduction

Dental caries and periodontal disease, as well as the possible sequel of both (tooth loss), are major dental public health problems worldwide. Poor oral health and untreated oral diseases have a negative impact on the quality of life of individuals and their overall health. This oral disease burden is markedly higher in developing countries like Mexico [1,2,3,4,5]. Dental caries is a multifactorial infectious and transmissible disease involving an imbalance of normal molecular interactions between the tooth surface/subsurface and the adjacent microbial biofilm where acids are produced [6]. Periodontal disease is a chronic bacterial infection that affects both the gingiva and the bone that supports the teeth; it is caused by anaerobic Gram-negative microorganisms that are present in the bacterial plaque that adheres to the teeth [7]. According to The Global Burden of Disease (GBD) 2010 Study, poor oral conditions remained highly prevalent in 2010, collectively affecting 3.9 billion people. The most common and the greatest burden globally is untreated caries in permanent teeth (global prevalence of 35% for all ages combined), whereas severe periodontitis and untreated caries in deciduous teeth were the 6th and 10th most prevalent conditions, affecting, respectively, 11% and 9% of the global population. Severe tooth loss was the 36th most prevalent condition, with a global estimate of 2% [3]. These diseases are equally highly prevalent and incident in Mexico across various age groups, and are concentrated in those with greater social disadvantage. A diet rich in simple carbohydrates, poor dietary patterns, inappropriate oral hygiene behaviors, restricted access to dental services, tobacco use, and excessive use of alcohol are some risk factors for these diseases [8,9,10,11,12,13,14,15,16,17,18,19,20,21,22,23,24].

Tobacco and alcohol use have considerable impacts on health [25,26,27,28,29,30], being two of the most important lifestyle and addiction considerations in this area. Tobacco use and tobacco smoke exposure remain the leading risk factors of preventable death globally. In Mexico, 21.7% of the 12 to 65 years age group are current smokers (31.4% male and 12.6% female), while 51.9% of the population has never smoked [31]. On the other hand, 71.3% of that population has used alcohol at some point in their lives (80.6% of men and 62.6% of women) [32]. There is considerable epidemiological evidence demonstrating the adverse effects of both tobacco and alcohol contributing to periodontal disease, tooth decay, tooth loss, staining of teeth and dental restorations, reduction of the ability to smell and taste, and oral conditions such as smoker’s palate, smoker’s melanosis, coated tongue, precancerous lesions and cancer, dental implant failures, and possibly oral candidosis [10,15,16,17,18,19,20,21,22,23,24]. In Mexico there are few studies that have addressed oral health conditions at the national level. The aim of this study was to determine whether use of tobacco and alcohol were associated with the prevalence of self-reported oral / dental problems in Mexican adults (18 and older).

## 2. Material and Methods

**Figure 1:**
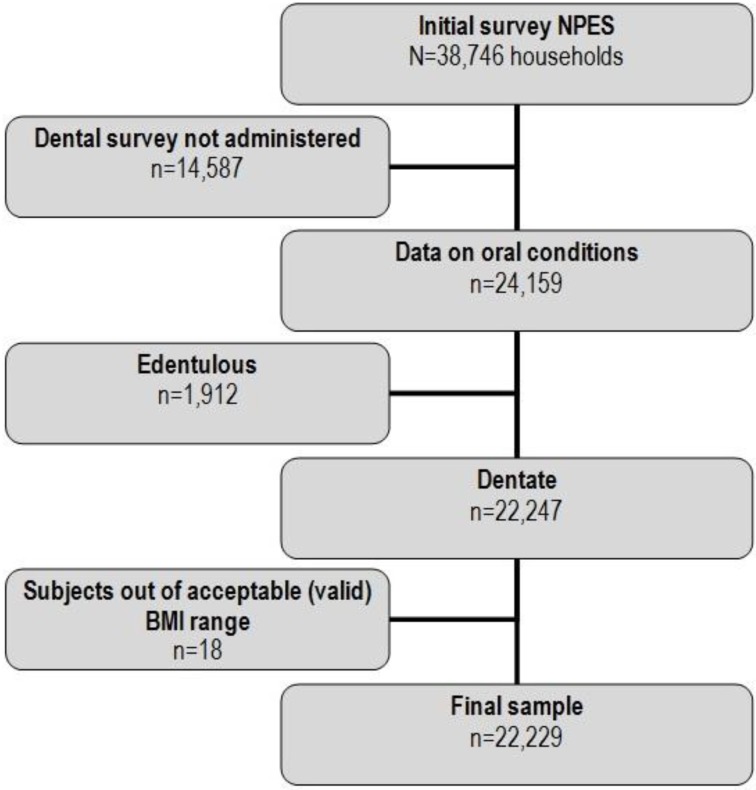
Flow chart for the individuals included in the study.

The present study conducted analyses of the National Performance Evaluation Survey 2003 (NPES) in Mexico. This cross-sectional study was part of the technical collaboration between the Ministry of Health and the World Health Organization (WHO), which used the survey instrument and sampling strategies developed by WHO for the World Health Survey (WHS). Further details of the survey methodology and on some oral health indicators are available elsewhere [33,34,35,36]. Information was collected from 38,746 households, with a mean of 1,250 households for each State. The sample design was probabilistic, multistage, stratified, through conglomerates, and was calculated to provide representative information at the State level, and across urban and rural areas. Data on dental conditions are available for only 20 out of the 32 States of Mexico (not every state implemented the entire set of survey modules), leading to a total of 24,159 households. Only subjects with teeth were included in the present analysis, so the final sample comprised 22,229 participants (Figure 1).

NPES consisted of two different questionnaires. In the first questionnaire, information was gathered with regard to residential services, income, expenses, and health insurance. The second questionnaire gathered information including health status, health risk factors, prevalence of key diseases, use of health services, non-clinical expectations of the population, and insurance coverage of certain clinical services. 

### 2.1. Dependent Variable

Self-reporting of oral/dental problems in the recent past was our dependent variable; it was gathered through question *Q6757* “*During the last 12 months*, *did you have any problems with your mouth and/or teeth?*”; the response options were *yes* or *no*.

### 2.2. Independent Variables

A series of socio-demographic and socio-economic factors and variables related to health were included: age (18 to 98 years), sex (male or female), residence (rural or urban), marital status (single, married, divorced, widowed, cohabitating), Indian ethnicity status (“*Do you speak an indigenous language?*”: *no* or *yes*), schooling (less than elementary school, complete elementary, complete middle school, high school/equivalent, college studies/higher), occupation (employed in the public sector, employed outside the public sector, self-employed, or not working or doing volunteer work), health insurance (insured or non-insured), socio-economic level (in tertiles), having a disability (none or yes), physical activity (high activity or low activity), chronic disease (none or any), body mass index (BMI; underweight <18.5, normal 18.5–24.9, overweight 25.0–29.9, or obesity ≥30), tobacco current use (never/not currently, sometimes, or daily) and alcohol current use (Never and low: fewer than 4 servings for women and 5 for men in the last week on one occasion; or high: 4 servings and more for women, and 5 for men in the last week on one occasion). Valid data were included as those BMI values between 10 and 58, and height measurements between 130 and 200 cm [37].

The household survey included general topics, such as building materials of the household and ownership of consumable goods, which were used to construct a Wealth Index (WI) using principal components analysis (PCA). The WI included owning a refrigerator, washing machine, dishwasher, personal computer, car, bicycle, television, *etc*., where goods were combined into a single estimator [38]. There were some missing data for weight (*n* = 326), height (*n* = 521), Indian ethnicity status (*n* = 196), health insurance (*n* = 48), and socio-economic level (*n* = 1), which were imputed through regression imputation [39].

First, we conducted a univariate analysis, reporting the summary measures according to each case for the nominal and ordinal variables (frequency and percentages) and the continuous variables (measures of central tendency and dispersion). A binary logistic regression analysis was used for the bivariate and multivariate analyses. The variance inflation factor was used to analyze and avoid multicollinearity between the independent variables. Variables with a statistical significance of *p* < 0.25 in the bivariate analysis were included in the final model. Subsequently, the *p*-values were calculated, and the odds ratios (OR) were calculated with a confidence interval (CI) of 95% and interactions were tested [40]. Due to the design used in the survey sampling, a module for complex samples was used for the statistical analysis (STATA 11.0, StataCorp, College Station, TX, USA).

The Medical Research Committee of the National Institute of Public Health in Mexico granted ethical approval. Participation in the survey was voluntary. All individuals provided written, informed consent.

## 3. Results

The study included 22,229 individuals, who were a weighted representation of 51,155,740 of the population in the country. Table 1 shows the descriptive results of several sociodemographic and socioeconomic variables, including both crude and weighed percentages. The 18–30 year olds age group made up 37.0% of the survey participants. Women accounted for 57.7% and those who lived in urban/metropolitan locations were 73.5%. Also, 56.9% of respondents were married and 8.5% were classified as being of indigenous ethnicity. As for the variables pertaining to socioeconomic status, 46.7% had completed middle school or higher, 50.2% had no formal employment, and 65.4% did not have any health insurance. The Wealth Index (WI) was divided into quartiles. Equally, in Table 1 we present several health-related variables for the study population: 4.5% reported having diabetes, 45.7% had a BMI in the normal/moderate range, 67.6% reported their general health as *very good*/*good*, while 21.7% and 46.3% reported using tobacco and alcohol currently, respectively. The prevalence of self-reported oral problems in the 12 months preceding the survey was 25.7%.

**Table 1:** Descriptive characteristics of the study population and prevalence of self-reported oral and dental problems (estimated national population *N* = 51,155,740) (National Performance Evaluation Survey 2003).

Variable	n	Percentage	N	% Weighted
***Age***				
18–30 years	7,676	34.5	18,912,188	37.0
31–45 years	7,600	34.2	18,256,912	35.7
46–and more years	6,953	31.3	13,986,640	27.3
***Sex***				
Male	9,397	42.3	21,655,360	42.3
Female	12,832	57.7	29,500,380	57.7
***Residence***				
Rural	6,462	29.1	13,544,848	26.5
Urban/Metropolitan	5,767	70.9	37,610,892	73.5
***Marital-status***				
Single	4,113	18.5	10,591,127	20.7
Married	12,836	57.7	29,118,247	56.9
Divorced	1,232	5.5	2,469,875	4.8
Widowed	1,570	7.1	2,631,745	5.1
Cohabitating	2,478	11.1	6,344,746	12.4
***Indian ethnicity status***				
No	20,817	93.6	46,822,650	91.5
Yes	1,412	6.4	4,333,090	8.5
***Schooling***				
Less than elementary	2,924	13.1	6,196,688	12.1
Complete elementary	9,393	42.3	21,082,147	41.2
Complete secondary	4,974	22.4	11,854,773	23.2
High School/equivalent	3,154	14.2	7,927,560	15.5
College and higher	1,784	8.0	4,094,572	8.0
***Occupation***				
Government Employee	2,212	9.9	4,297,865	8.4
Non-Government Employee	3,030	13.6	7,358,914	14.4
Self-Employed	5,796	26.1	13,494,269	26.4
Employer	58	0.3	87,830	0.2
Voluntary Worker	96	0.4	235,224	0.5
Does not work	11,037	49.7	25,681,638	50.2
***Health insurance***				
Non-insured	14,129	63.6	33,435,760	65.4
Social security	7,561	34.0	16,433,454	32.1
Other insurance	539	2.4	1,286,526	2.5
***Wealth Index***				
1 quartile (lowest)	5,559	25.0	12,912,573	25.2
2 quartile	5,573	25.1	11,981,913	23.4
3 quartile	6,149	27.7	13,754,179	26.9
4 quartile (highest)	4,948	22.2	12,507,075	24.4
***Diabetes***				
No	21,223	95.5	48,862,029	95.5
Yes	1,006	4.5	2,293,711	4.5
***BMI***				
Underweight < 18.5	544	2.4	1,153,652	2.3
Normal BMI (18.5–24.9)	9,883	44.5	23,357,437	45.7
Overweight (25.0–29.9)	8,394	37.8	19,345,482	37.8
Obesity (≥30)	3,408	15.3	7,299,169	14.3
***Self-reported health***				
Very good/good	14,709	66.2	34,602,142	67.6
Moderate	6,366	28.6	14,163,997	27.7
Bad/very bad	1,154	5.2	2,389,601	4.7
***Smoking (current)***				
No	17,555	79.0	40,029,133	78.2
Sometimes	3,095	13.9	7,731,460	15.1
Daily	1,579	7.1	3,395,147	6.6
***Alcohol Use (current)***				
No	11,724	52.7	27,446,858	53.7
Low	9,976	44.9	22,716,583	44.4
High	529	2.4	992,299	1.9

Note: **SRODP** = Self-report of oral and dental problems.

Table 2 shows the prevalence of reported oral problems across the categories of the independent variables included. In the bivariate logistic regression analysis (Table 2) all variables were significant (*p* < 0.05), except for education and occupation.

**Table 2:** Bivariate analyses of the prevalence of self-report of oral and dental problems and independent variables (National Performance Evaluation Survey 2003).

Variable	% SRODP	OR	95% CI	*p* value *
***Age***				
25 years	18.9	1 *		
25–44 years	23.8	1.33	1.15–1.54	<0.001
45–64 years	33.2	2.12	1.82–2.49	<0.001
65 and more years	33.7	2.17	1.78–2.64	<0.001
***Sex***				
Male	22.6	1 *		
Female	27.9	1.32	1.20–1.46	<0.001
***Residence***				
Rural	21.5	1 *		
Urban/Metropolitan	27.1	1.25	1.21–1.50	<0.001
***Marital-status***				
Single	22.0	1 *		
Married	26.0	1.24	1.09–1.42	0.001
Divorced	33.0	1.74	1.37–2.22	<0.001
Widowed	33.5	1.78	1.44–2.20	<0.001
Cohabitating	23.8	1.11	0.92–1.33	0.268
***Indian ethnicity status***				
No	26.1	1 *		
Yes	21.2	0.76	0.63–0.91	0.004
***Schooling***				
Complete elementary & less	25.2	1 *		
Complete secondary	25.4	1.01	0.89–1.14	0.874
High School/equivalent	25.3	1.00	0.87–1.15	0.958
College and higher	29.7	1.24	1.04–1.49	0.014
***Occupation***				
Government Employee	25.7	2.10	0.80–5.51	0.131
Non-Government Employee	22.6	1.77	0.67–4.64	0.242
Self-Employed	24.9	2.01	0.77–5.24	0.152
Employer	14.1	1 *		
Voluntary Worker	30.5	2.66	0.84–8.38	0.093
Does not work	26.9	2.23	0.86–5.80	0.099
***Health insurance***				
Non-insured	24.3	1 *		
Social security	27.7	1.19	1.07–1.31	0.001
Other insurance	35.1	1.68	1.26–2.24	<0.001
***Wealth Index***				
1 quartile (lowest)	20.5	1 *		
2 quartile	24.1	1.23	1.07–1.41	0.003
3 quartile	27.1	1.44	1.26–1.65	<0.001
4 quartile (highest)	30.9	1.74	1.51–1.99	<0.001
***Diabetes***				
No	24.7	1 *		
Yes	45.6	2.54	2.06–3.14	<0.001
***BMI***				
Underweight < 18.5	17.4	0.67	0.48–0.92	0.014
Normal BMI (18.5–24.9)	23.9	1 *		
Overweight (25.0–29.9)	26.3	1.13	1.01–1.26	0.023
Obesity (≥30)	30.7	1.41	1.22–1.62	<0.001
***Self-reported health***				
Very good/good	21.5	1 *		
Moderate	33.2	1.81	1.63–2.01	<0.001
Bad/very bad	41.3	2.56	2.08–3.16	<0.001
***Smoking (current)***				
No	22.8	1 *		
Sometimes/Daily	29.0	1.27	1.13–1.43	<0.001
***Alcohol Use (current)***				
No	24.6	1 *		
Low/High	29.4	1.38	1.25–1.52	<0.001

Notes: **SRODP** = Self-report of oral and dental problems; * The Pearson chi-squared statistic is corrected for the survey design using the second-order correction of Rao and Scott and converted into an F-statistic.

Finally, three logistic regression multivariate models were fitted, one for each age group (Table 3). Women were 47%, 48%, and 29% (*p* < 0.05) more likely to report oral problems than men for each age group, respectively. We also found some differences associated with variations in socioeconomic status, except for those survey participants in the 46–98 years old age group: subjects with higher educational attainment (18–30 years: OR = 1.40; 95% CI = 1.23–1.59, and 31–45 years: OR = 1.17; 95% CI = 1.04–1.31) compared with those with less education; and those who were scored higher in the Wealth Index (4th quartile OR = 1.26, OR = 1.18, OR = 1.23 for each age group) compared to those in worst Wealth Index quartiles (1st, 2nd and 3rd), had higher odds of presenting oral/dental problems (*p* < 0.05).

With regard to the variables related to general health we noted that subjects with diabetes had higher odds of self-reporting oral/dental problems in the 12 months preceding the study (*p* < 0.05), except for the 18–30 years age group (*p* > 0.05). Also, individuals who reported having *moderate* and *bad/very bad* general health were more likely to have oral/dental problems (*p* < 0.01) than individuals who reported having *very good*/*good* health, across all age groups.

As far as tobacco use was concerned, in the 18–30 years age group no significant differences were found for oral/dental conditions (*p* > 0.05). However, for the 31–45 and 46–98 years age groups the odds ratios of reporting oral/dental conditions were greater among those who admitted to using tobacco (sometimes or daily) (OR = 1.24; 95% CI = 1.08–1.42 and OR = 1.16; 95% CI = 1.01–1.32, respectively) than those who reported not using tobacco. As far as alcohol use was concerned, there were significant differences (*p* < 0.05) in the three age groups. The odds ratios of reporting oral/dental problems were greater among those who used alcohol (low or high) compared to those who denied using alcohol (OR = 1.26; *p* < 0.001, OR = 1.18; *p* < 0.01, and OR = 1.30; *p* < 0.001, respectively for each age group).

**Table 3:** Results of multivariate logistic regression models for each one of the three age groups on the prevalence of self-reported oral and dental problems and independent variables (National Performance Evaluation Survey 2003).

Variable	OR (95% CI) 18–30 years	OR (95% CI) 31–45 years	OR (95% CI) 46–98 years
***Sex***			
Male	1 *	1 *	1 *
Female	1.47 (1.30–1.67) **^†^**	1.48 (1.31–1.66) **^†^**	1.29 (1.16–1.45) **^†^**
***Schooling***			
Complete elementary & less	1 *	1 *	1 *
More than elementary school	1.40 (1.23–1.59) **^†^**	1.17 (1.04–1.31) **^‡^**	1.11 (0.96–1.28) n/s
***Wealth index***			
1, 2, 3 quartile	1 *	1 *	1 *
4 quartile (highest)	1.26 (1.09–1.45) **^‡^**	1.18 (1.03–1.35) ^¶^	1.23 (1.08–1.40) **^‡^**
***Diabetes***			
No	1 *	1 *	1 *
Yes	1.00 (0.50–1.99) n/s	1.63 (1.21–2.19) **^ ‡^**	1.54 (1.32–1.81) **^†^**
***Self-reported health***			
Very good/good	1 *	1 *	1 *
Moderate	1.95 (1.71–2.23) **^†^**	1.79 (1.60–2.01) **^†^**	1.47 (1.31–1.64) **^†^**
Bad/very bad	1.73 (1.20–2.48) **^‡^**	2.29 (1.79–2.94) **^†^**	1.85 (1.56–2.21) **^†^**
***Smoking (current)***			
No	1 *	1 *	1 *
Sometimes/Daily	1.15 (0.99–1.34) n/s	1.24 (1.08–1.42) ^‡^	1.16 (1.01–1.32) ^¶^
***Alcohol Use (current)***			
No	1 *	1 *	1 *
Low/High	1.26 (1.11–1.43) ^†^	1.18 (1.05–1.33) ^‡^	1.30 (1.16–1.45) ^†^

Notes: * Reference category; **^†^** *p* < 0.001; **^‡^** *p* < 0.01, ^¶^ *p* < 0.05.

## 4. Discussion

This study was undertaken to determine whether the use of alcohol and tobacco were associated with the prevalence of reported oral/dental problems in Mexican adults with teeth, using data from a national representative survey. Although oral health is an essential component of overall health and wellbeing, and treatment of oral diseases is expensive and poses a financial impact on families and society, oral health does not always hold an important place in the priorities of individuals or healthcare systems [3,4]. From an epidemiological point of view, the study of oral health needs across diverse population groups can help to focus human and financial resources toward solving health problems that occur in the general population [41]. We found an overall prevalence of self-reported oral/dental problems of 25.7% in the 12 months preceding the study. In South Africa, 16% of respondents reported having had problems with their mouth and/or teeth [42], however the study used a reference period of six months for self-reporting.

We observed that tobacco and alcohol use were associated with self-reported oral/dental problems. Excessive consumption of tobacco and alcohol are considered serious public health problems in Mexico and around the world due to their high social and economic costs; also because some perspectives argue these substances are the first entry points to multiple risk behaviors. Their overall health impacts in terms of morbidity, disability and mortality include not only harm but also health care direct costs and indirect costs due to inability, disability and loss of production; they also entail intangible costs such as suffering, pain and family impacts [31,32,43]. In relation to oral health, several studies have linked excessive use of these substances with oral diseases such as caries, periodontal disease, and dental needs: our results are partially consistent with those findings [15,16,17,18,19,20,21,22,23,24,33], emphasizing that these two substances may be associated with worse oral health status. Given the format of the questions in the national survey it is important to note that excessive use in the prior week does not necessarily equate to addiction. There are multiple hypothesized links, such as the suggestion that smoking can promote caries by fostering increased formation of *S. mutans* biofilm on tooth surfaces [44]. There is sufficient evidence to infer a causal relationship with smoking for cancers in the oral cavity and pharynx, as well as periodontitis. Evidence is suggestive but not sufficient to infer a causal relationship between smoking and root-surface caries and between maternal smoking and oral clefts. Furthermore, a negative response to periodontal treatment has consistently been reported [45,46]. The most obvious biological connection between substances in tobacco smoke and oral/dental health is the destruction of tooth-supporting tissue [47]. The destructive influences of smoking on the periodontal tissues span from suppression of inflammation through interference with vascular and immune reactions to undermining bone support, leading to bone loss, pocket formation, and tooth loss [16]. In relation to alcohol use, one of the effects may be increased cariogenic potential derived from sweet drinks high in simple sugars and acid content in alcoholic drinks. Additionally, combined with a cariogenic, nutritionally poor diet, poor oral hygiene measures, decreased salivary flow, and a high incidence of smoking in those persons with high alcohol intake, alcohol use may provide an environment conducive to rapid progression of periodontal disease and caries [48]. Another mechanism contributing to this link could be lower utilization of health services generally by persons who use alcohol (in particular those who abuse it) since it is reasonable to propose that high risk behaviors and low utilization of preventive/curative services lead to higher and earlier morbidity [49]. As members of the health team, dentists have an obligation to promote oral health and healthy lifestyles among their patients, and this responsibility includes increasing awareness of the harmful effects of alcohol and tobacco on general health and oral health [50]. Although estimators for tobacco and alcohol use were different, confidence intervals suggested differences were not significant. It seems more salient the individual effects on oral/dental health. 

Epidemiologic evidence worldwide confirms the relationship between socioeconomic status and oral health of individuals, regardless of the indicators used; those with higher status have better oral health, a feature known as social health gradient. While most studies have found this effect in clinical settings, the linkage with self-reported health measurements is not entirely consistent [51,52]. There are multiple possible explanations for this contradictory finding: (1) although individuals with more social disadvantage have worse oral health conditions, those with better socioeconomic position could better identify oral health needs through enhanced access to information and health education. On the other hand, (2) people with worse socioeconomic position may have more urgent priorities in life and oral health may not be one of them; under this interpretation, symptoms associated with oral diseases could not represent “meaningful” health needs. Finally, (3) it has been observed that people of lower socioeconomic position are less likely to receive dental care even in the presence of pain [53] and this trend could be primarily due to barriers in access to care. Many complex relationships undoubtedly moderate the interactions between variables.

Socio-demographic variables such as age and sex have also been linked to health status; as age increases, disease experience also increases, just as in oral health needs. It has been found that women in Mexico have oral health and higher oral health needs [33]. In terms of health, the variables of sex (biological construct) and gender (behavioral and social construct) are recognized as useful parameters for research and action, as differences determine specific diseases for men and for women [54]. In this regard, the observed differences between men and women have been well documented by other authors in several health topics [55], as well as in our own study, with women reporting worse health status than men. One of the main explanations for such differential is that women may recognize pain and discomfort more easily than men [55]. Other explanations that have been proposed are (1) the specific conditions of women (maternal conditions, risk exposures, poverty and social exclusion, empowerment), especially in developing countries; (2) conditions associated with increased longevity in women (arising from aging and chronic diseases); (3) conditions resulting from the interaction of sex and gender (depressive symptoms); and (4) gender-based conditions (violence, for example) [54]. Finally, although our study was based on self-report of oral health/dental problems, our findings agree with previous observations in which subjects with diabetes have poor oral health, manifested especially as periodontal disease, tooth loss and caries [33,56,57].

The limitations of the present study are mainly related to its design; a cross-sectional study, in which the cause and the effect are measured at the same time, emphasizes the need to avoid making extrapolations to other population groups because we cannot distinguish between cause and effect in our observations. Another limitation is the lack of clinical confirmation by normative evaluations of dental and periodontal disease, which would have provided a more detailed investigation rather than solely relying on self-reported health conditions. It is also possible that our results may be somewhat dated, as the survey accrued information from several years ago and hence disease profiles may have suffered some changes. However, to our knowledge, this is the first study conducted in Mexico and Latin America based on the World Health Survey data using this indicator as a response variable. Based on the results we conclude that one out of four adults in Mexico reported having had oral problems on the 12 months prior to the study data collection. Tobacco and alcohol use were associated with increased prevalence of oral disease. Besides its confirmatory value using national level data, in the larger scheme of things our results lend further credence to the need to adopting measures to reduce consumption of tobacco and alcohol for the specific purpose of improving oral health. This approach would be a separate consideration from implementing an overall common risk factor approach to support public health and health promotion measures to reduce excessive use of tobacco and alcohol, in order to improve both general health and oral health outcomes.
